# Accelerating Wound Healing Through Deep Reinforcement Learning: A Data-Driven Approach to Optimal Treatment

**DOI:** 10.3390/bioengineering12070756

**Published:** 2025-07-11

**Authors:** Fan Lu, Ksenia Zlobina, Prabhat Baniya, Houpu Li, Nicholas Rondoni, Narges Asefifeyzabadi, Wan Shen Hee, Maryam Tebyani, Kaelan Schorger, Celeste Franco, Michelle Bagood, Mircea Teodorescu, Marco Rolandi, Rivkah Isseroff, Marcella Gomez

**Affiliations:** 1 Applied Mathematics, Baskin Engineering, University of California Santa Cruz, Santa Cruz, CA 95060, USA; 2 Electrical and Computer Engineering, Baskin Engineering, University of California Santa Cruz, Santa Cruz, CA 95060, USA; 3 Biomedical Engineering, University of Southern California, Los Angeles, CA 90089, USA; 4 Bioengineering Department, Clemson University, Clemson, SC 29634, USA; 5 Department of Dermatology, University of California Davis, Davis, CA 95616, USA

**Keywords:** wound healing, deep reinforcement learning, biomedical engineering

## Abstract

Advancements in bioelectronic sensors and actuators have paved the way for real-time monitoring and control of the progression of wound healing. Real-time monitoring allows for precise adjustment of treatment strategies to align them with an individual’s unique biological response. However, due to the complexities of human–drug interactions and a lack of predictive models, it is challenging to determine how one should adjust drug dosage to achieve the desired biological response. This work proposes an adaptive closed-loop control framework that integrates deep learning, optimal control, and reinforcement learning to update treatment strategies in real time, with the goal of accelerating wound closure. The proposed approach eliminates the need for mathematical modeling of complex nonlinear wound-healing dynamics. We demonstrate the convergence of the controller via an in silico experimental setup, where the proposed approach successfully accelerated the wound-healing process by 17.71%. Finally, we share the experimental setup and results of an in vivo implementation to highlight the translational potential of our work. Our data-driven model suggests that the treatment strategy, as determined by our deep reinforcement learning algorithm, results in an accelerated onset of inflammation and subsequent transition to proliferation in a porcine wound model.

## 1. Introduction

Wound healing is a dynamic and continuous process that involves nonlinear interactions across different cell types (platelets, neutrophils, macrophages, myofibroblasts, fibroblasts, keratinocytes, and others) and bio-molecules (blood coagulation factors, pro- and anti-inflammatory cytokines, polymers, and enzymes of the extracellular matrix) [1]. These nonlinear processes can be modulated by different treatments administrated to the wound, which thus influence the healing process. In this context, personalized precision treatments have emerged as a vital research area in modern health care, driven by recent advancements in artificial intelligence [2,3,4]. The need for precision treatment arises from the fact that patients often exhibit varied responses to the same medication, a phenomenon that arises from both molecular differences between individuals and variations within the same patient over time [5]. Personalized treatment aims to identify the most effective drug types, dosages, and timings of administration for each patient’s unique responses, leveraging experimental data and statistical analysis [6].

In this work, we explore two treatment modalities: electric field (EF) therapy and fluoxetine (Flx), both of which have demonstrated efficacy in accelerating wound healing. Endogenous electric fields facilitate healing by promoting the migration of epidermal stem cells, which are vital for tissue repair [7]. Previous studies have shown that fluoxetine, when administered either systemically or topically, enhances wound healing in both diabetic and non-diabetic rodent models [8]. Moreover, fluoxetine has been found to inhibit pathogen growth, reduce biofilm formation, and limit the spread of infection in rodents [9]. More recent work confirms that fluoxetine suppresses inflammation and, as a consequence, can delay healing if applied too early [10,11,12]. Given this prior knowledge, our high-level strategy is prescriptive in that we initiate treatment with EF and then switch to fluoxetine as soon as sufficient levels of inflammation are detected. However, determining the optimal strength of the electric field and the optimal fluoxetine dosage presents a significant challenge, particularly when accounting for the nonlinear dynamics of cell migration induced by electric fields, drug metabolism, and the biological mechanisms targeted by the medication.

While mathematical models can help inform optimal treatment strategies [10,13], the complexity of biological systems and individual variability can undermine the reliability of model-based controllers. Even with accurate models, the inherent nonlinearity complicates the task of ensuring the optimality and safety of prescribed treatment strategies for wound care. Conversely, the study of linear systems is well established, providing scalable methods for designing, analyzing, controlling, and optimizing such systems [14,15]. Thus, we aim to learn a mapping from a nonlinear system to a linear one. Establishing a reliable mapping from nonlinear systems to linear models remains challenging. The Koopman operator theory, as discussed in foundational works [16,17,18], offers a promising approach by allowing a nonlinear system to be represented as an infinite-dimensional linear system. Yet, the optimization of this framework typically operates within functional space, which can be impractical in real-world applications and does not account for control inputs in nonlinear systems. Recent advances have sought to generalize Koopman operator theory to the control of nonlinear systems [19,20,21,22]. These extensions provide finite-dimensional function approximations that can facilitate the tractable control of nonlinear systems. This motivated us to design a deep neural network-based algorithm to learn a mapping from an unknown nonlinear system to its linear representation, an operation akin to the objectives of the Koopman operator. Our work builds upon previous research [21,22,23]. The major difference is that our approach addresses overfitting during training and does not rely on prior knowledge of a control–affine matrix. This work is coupled with optimal control and deep reinforcement learning (DRL) to optimize treatment strategies.

**Figure 1:**
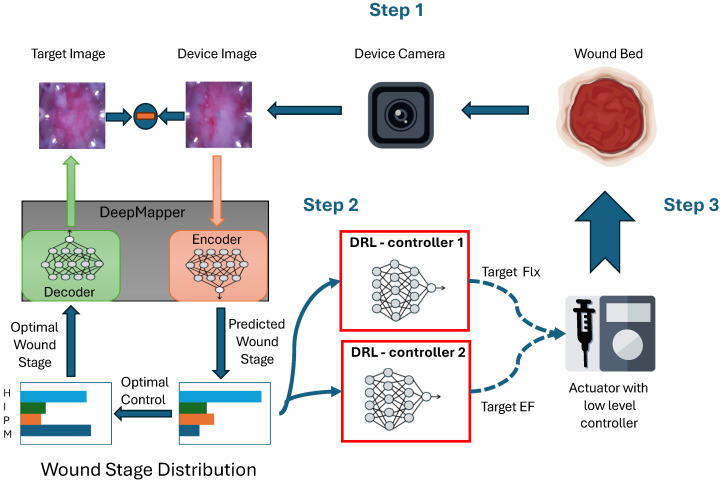
The DRL-based closed-loop control pipeline for accelerating wound healing involves three steps. First, a wound image captured by a camera is fed into DeepMapper, which predicts the wound stage. The following four canonical wound stages are represented: hemostasis (H), inflammation (I), proliferation (P), and maturation (M). Second, the deep reinforcement learning (DRL) agents generate treatment strategies. Third, an electric field or a dose of fluoxetine is delivered to the wound through an actuator.

The main contribution of this paper is an adaptive closed-loop control framework using deep learning, optimal control, and reinforcement learning to enhance wound healing, as schematized in Figure 1. At the heart of the algorithm is DeepMapper, which provides an invertible mapping between a nonlinear state and a linear state. In this case, the nonlinear system is the temporal evolution of wound images, which are mapped to a linear system with four states representing the four canonical stages of wound healing: hemostasis (H), inflammation (I), proliferation (P), and maturation (M). DeepMapper also generates a linear ordinary differential equation that models the temporal evolution of these four wound-healing stages. The linear model is used to derive an optimal control law and the corresponding target nonlinear state (target image) to guide the deep reinforcement learning algorithm.0 That is, the error between the device and target image is used to update the deep reinforcement learning algorithm and adjust the applied EF strength or dosage of fluoxetine. The response of the wound to the treatment is captured in the next image, and the cycle repeats. This framework eliminates the need for mathematical modeling of nonlinear dynamics or the mechanism by which a treatment affects the wound-healing processes.

We generalized the DeepMapper method from [23], eliminating the need for a control–affine matrix. Our results demonstrate its ability to learn a linear representation of nonlinear wound-healing dynamics from a time-series of images monitoring wound closure in vivo. By leveraging optimal control and the decoder mechanism within DeepMapper, we derived an optimal reference signal that the DRL agent used to generate real-time treatment strategies. This approach not only improved the accuracy of the linear representation for modeling wound dynamics under optimal treatments but also enhanced the efficiency of the reinforcement learning agent. We validated the efficacy of our approach with experimental data. Simulations of nonlinear healing dynamics with adaptive interventions showed that the proposed method accelerated wound healing by 17.71% compared to the normal healing process. Additionally, the proposed framework has successfully been implemented in in vivo porcine experiments, demonstrating our method’s potential for translation to clinical settings [24]. The proposed framework showcases the significant potential to expedite wound healing by effectively integrating optimal control and data-driven methods. By leveraging advanced algorithms that adapt in real time to changing conditions, this system offers a more accurate and reliable means of promoting faster recovery that lacks the limitations of conventional models.

The remainder of the paper is organized as follows. Section 2 presents an overview of the closed-loop control framework and then the details of the design of the deep learning-based algorithm used to find the linear representation of nonlinear wound-healing dynamics, as well as a DRL-based algorithm for accelerating wound healing. Implementation details, simulation results, and a detailed discussion of these results are given in Section 3 and Section 4. Finally, in Section 5, we present conclusions and future research directions.

## 2. Materials and Methods

The control and optimization of wound-healing dynamics, particularly when one is devising the most effective treatment strategy, is challenging due to their inherently nonlinear nature. Previous work [21,22,23] has focused on finding linear representations of nonlinear systems from which optimal control can be derived. However, these approaches often require detailed knowledge of the control effects on the learned linear systems, such as a control–affine matrix, as in [23]. In practice, obtaining this information is often difficult, making it challenging to translate the learned linear system into actionable treatment parameters such as drug dosage or the strength of the electric field.

To address this gap, we employ a leader–follower strategy that is commonly used in robotic control by leveraging a decoder alongside a deep reinforcement learning (DRL) controller. This is made possible by DeepMapper, which both learns a linear representation of the nonlinear system and uses it to compute an optimal control strategy, bridging the gap between theoretical models and practical treatment parameters.

### 2.1. DeepMapper: Linearization of Nonlinear Wound-Healing Dynamics

Consider a nonlinear state-space model in discrete time, as follows:

(1) $$x_{k+1}=F\left(x_{k},u_{k}\right),\;k\geq 0,\;\;x_{0}=x\in X.$$

The state space X is a closed subset of ${ℜ}^{n}$, and the input (or action) space U is finite, with cardinality $n_{U}:=\left|U\right|$, where F:X×U→X. We may have state-dependent constraints, so that for each x∈X. there is a set U(x)⊂U for which $u_{k}$ is constrained to $U(x_{k})$ for each k.

Assume that there exists one unique equilibrium $x^{e}$, which represents the full closure of the wound. This paper concerns finding an optimal control strategy $u^{★}\in \phi^{★}\left(x\right)$ such that the time from any initial state *x* to an equilibrium $x^{e}$ is minimized.

As discussed in [10], the function *F* can be an unmanageable nonlinear function that defines different cell transitions during wound healing. The nonlinear state *x* associated with wound healing as surveyed in the literature may include variables such as pH, temperature [25,26,27,28,29,30], or visual representations captured through images of the wound [31]. We assume that *x* can be measured by some sensor, but *F* is unknown to the control algorithm.

Solving for the optimal control policy $u^{★}\in \phi^{★}\left(x\right)$ is often difficult, particularly when the dynamics evolve nonlinearly. Given that extensive research and literature has been dedicated to the study of linear systems, encompassing scalable design, analysis, control, and optimization [14,32], we propose the utilization of a deep learning approach to model a linear system that best approximates the behavior of the underlying nonlinear system to guide control efforts. This builds on our previous work [23] to create a more generalized approach. The major differences are the following: 1. in this work, we do not assume knowledge of a control–affine matrix; 2. we formulate the problem in discrete time, thus removing the need to calculate the Jacobian matrix of a deep neural network and improving the efficiency of the algorithm.

Note that (1) defines a control–affine system. As discussed in [10,21], the decoupling of the states and inputs allows us to find a transformation of the states alone, as follows:

(2) z=h(x)

where $x\in {ℜ}^{n}$ is the state that evolves subject to nonlinear dynamics, $h:{ℜ}^{n}\to {ℜ}^{d}$ is a function that maps the nonlinear state *x* to linear state *z*, and $z\in {ℜ}^{d}$ evolves linearly, as follows:

(3) $$\begin{array}{cc} z_{k+1} & =A^{nat}z_{k}+A^{act}\nu_{k},\;k\geq 0\\ A^{\mathrm{nat}} & =\left[\begin{array}{cccc} -k_{h}^{nat} & 0 & 0 & 0\\ k_{h}^{nat} & -k_{i}^{nat} & 0 & 0\\ 0 & k_{i}^{nat} & -k_{p}^{nat} & 0\\ 0 & 0 & k_{p}^{nat} & 0 \end{array}\right],\\ A^{\mathrm{act}} & =\left[\begin{array}{cccc} -k_{h}^{act} & 0 & 0 & 0\\ k_{h}^{act} & -k_{h}^{act} & 0 & 0\\ 0 & k_{h}^{act} & -k_{h}^{act} & 0\\ 0 & 0 & k_{h}^{act} & 0 \end{array}\right], \end{array}$$

where $z_{0}:=z={\left[H,I,P,M\right]}^{⊤}\in {\left[0,1\right]}^{4}$, $A^{nat},A^{act}\in {\left[0,1\right]}^{4\times 4}$ contains values that control the velocities of transitions from *H* (homeostasis) to *I* (inflammation), from *I* to *P* (proliferation), and from *P* to *M* (maturation).

Assume that $A^{nat}+A^{act}\in {\left[0,1\right]}^{4}$; with initial condition $z={\left[1,0,0,0\right]}^{⊤}$, there exists one unique equilibrium in the linear dynamics: $z^{e}={\left[0,0,0,1\right]}^{⊤}$, which represents a fully healed wound (100% maturation). Without loss of generality, we assume that $z^{e}=h\left(x^{e}\right)$. Thus, minimizing the time taken from any initial state *x* to an equilibrium $x^{e}$ is equivalent to minimizing the following:

$$J=\sum_{k=0}^{\infty }\left(z_{k}^{⊤}Qz_{k}+\nu_{k}^{⊤}R\nu_{k}\right)$$

with $Q=\left[\begin{array}{cccc} 1 & 0 & 0 & 0\\ 0 & 1 & 0 & 0\\ 0 & 0 & 1 & 0\\ 0 & 0 & 0 & 0 \end{array}\right]$ and $R=\left[\begin{array}{cccc} 1 & 0 & 0 & 0\\ 0 & 1 & 0 & 0\\ 0 & 0 & 1 & 0\\ 0 & 0 & 0 & 1 \end{array}\right]$.

The optimal-control problem for the linear dynamics (3) can be solved via the discrete-time algebraic Riccati equation, giving the optimal control law as follows:

(4) $$\nu^{★}=-Kz,$$

which is referred to as the Linear Quadratic Regulator (LQR) [32].

The optimal control law in (4) informs only the transition rates between wound-healing stages required to minimize the time needed for maturation to reach 100%. However, it does not give any information on what treatments should be applied to the wound, such as what drug dosage should be used. Thus, we need a mechanism that can map the linear state *z* to the nonlinear state *x* to inform the real treatment *u*. In this paper, we adopted the updated DeepMapper architecture proposed in [33], where we employed three types of deep neural networks to parameterize the transformation function *h*, an inverse transformation function $h^{-1}:{ℜ}^{d}\to {ℜ}^{n}$, and the two matrices $A^{nat}$ and $A^{act}$ in (3).

In a system similar to that in [33], there are three high-level requirements for the neural networks, corresponding to the three types of loss function used in training:

1. *Intrinsic coordinates that are useful for reconstruction.* We seek to identify a few intrinsic coordinates z=h(x) where the dynamics evolve, along with the inverse $x=h^{-1}\left(z\right)$ so that the state *x* may be recovered. The reconstruction accuracy of the auto-encoder is achieved by minimizing the loss, as follows:

$$L_{1}=∥x_{k}-h^{-1}\left(h\left(x_{k}\right)\right)∥,\;\;\;k\geq 0.$$

with ∥·∥ as the mean-squared error, averaging over dimension then number of examples.

2. *Prediction of future states.* The intrinsic coordinates must enable prediction of future states. Specifically, we identified linear dynamics in the matrix $A:=A^{nat}-A^{act}K$. This corresponded to minimizing the loss, as follows:

$$\begin{array}{cc} L_{2}\left(\theta ,\omega \right) & =∥x_{k+1}-h^{-1}\left(Ah\left(x_{k}\right)\right)∥\\ \; & =∥F\left(x_{k},u_{k}\right)-h^{-1}\left(Ah\left(x_{k}\right)\right)∥,\;\;\;k\geq 0. \end{array}$$

3. *Linear dynamics.* To discover the mapping function *h*, we learned the linear dynamics *A* on the intrinsic coordinates, i.e., $z_{k+1}=Az_{k}$. Linear dynamics were achieved by minimizing the following loss, as below:

$$\begin{array}{cc} L_{3} & =∥h\left(x_{k+1}\right)-Ah^{\theta }\left(x_{k}\right)∥\\ \; & =∥h\left(F\left(x_{k},u_{k}\right)\right)-Ah\left(x_{k}\right)∥,\;\;\;k\geq 0. \end{array}$$

The final loss function is a weighted sum of $L_{1}$, $L_{2}$, and $L_{3}$, as follows:

(5) $$L=w_{1}L_{1}+w_{2}L_{2}+w_{3}L_{3}$$

where $w_{1},w_{2},w_{3}\in {ℜ}^{+}$ with $w_{1}+w_{2}+w_{3}=1$.

In biological systems, once a wound is created, the natural healing dynamics $A^{nat}$ remain relatively stable over time, while the external environment affecting the wound can change frequently. This necessitates the design of a model where $A^{nat}$ updates at a slower rate than $A^{act}$; we should also consider capturing the temporal relationship of the measurements $\left\{x_{k}\right\}$ and the delayed biological responses to changes in external conditions or treatments. Thus, $A^{nat}$ is updated every *N* samples and $A^{act}$ is updated through all the samples within a window of size *N* through the attention mechanism detailed in [33].

Note that minimizing the loss *L* in (5) gives only an estimation of the linear representation of the nonlinear wound-healing dynamics through which an optimal control law can be derived. To derive the real optimal treatment strategy $u^{★}\in \phi^{★}\left(x\right)$, in this paper, we propose the use of a DRL agent to track the reference signal incurred by (4) whenever it is available and penalize the DRL agent when it is not. We show that the control law learned by this DRL agent outperforms direct optimization over the nonlinear system without a learned mapping, achieving faster convergence and more stability.

For the in silico experiments, DeepMapper was trained on an augmented dataset originating from 256 wound images from mice. For in vivo experiments, the model was further trained on a collection of wound images from five in vivo experiments conducted on porcine models. Each of these porcine experiments contains a time series of images, with six images taken every 2 h for 3 days. Although this yielded approximately 900 images in total, we performed a downsampling step with each set of six images from each time point. This gave us a set of around 150 porcine images. For data augmentation, we conducted random rotation and added Gaussian noise.

### 2.2. Design of the Reinforcement Learning Algorithm

In this section, we introduce the use of a DRL algorithm to explore possible treatment policies such that when the optimal control input (4) is accessible, the DRL algorithm should be able to exploit its acquired knowledge to generate a policy that drives the linear state towards the one generated through optimal control. The exploration and exploitation of the DRL algorithm do not require knowledge of either nonlinear or linear dynamics, and thus, this approach not only alleviates the burden of mathematical interpretation in real-world treatment scenarios but significantly expedites the healing process. To realize this, we formulated the wound-healing dynamics as a Markov decision process (MDP) problem and subsequently solved it using the Advantage Actor Critic (A2C) algorithm [34].

Consider an MDP defined by (X,U,P,r,γ), where X represents the state space, U represents the input/action space, *P* represents the transition-probability matrix, *r* represents the reward function, and γ represents the discount factor. In MDP, an autonomous agent makes sequential discrete-time decisions as time passes. Generally speaking, the MDP problem conforms to the decision-making process of physicians in wound care. Based on the state $x_{k}\in X$, the agent selects action $u_{k}\in U$ at time *k*, then observes the next state $x_{k+1}$ and receives the reward $r(x_{k},u_{k})\in ℜ$. To collect more state information for wound management, the agent can perform state observation more frequently; for example, it may perform a state observation every hour and select an action every 20 min [10,31]. The state $x_{k}$ transits to the next state $x_{k+1}$ following the transition-probability matrix $P(x_{k+1}|x_{k},u_{k})$, which represents the dynamics of the operating environment. The transition-probability matrix satisfies the Markovian (or memoryless) property, since a transition to the next state $x_{k+1}$ depends only on the current state $x_{k}$ and action $u_{k}$, rather than on a historical series of states and actions. The agent learns the optimal policy $\phi^{★}:X\to U$, which maps x∈X to optimal actions u∈U over trial-and-error interactions with the environment. Nevertheless, the transition-probability matrix and the probability distribution of the reward function are generally unknown in reality.

This article designed the actions, states, and rewards based on the characteristics of wound healing. The action u∈ℜ in the algorithm indicates the EF (electric field) intensity or the dosage of the drug. The state *x* is an image of the wound. The reward *r* of the algorithm is the exponential of negative Euclidean distance between the next wound image and the image generated by the linear state, as follows:

(6) $$\begin{array}{c} r\left(x_{k},u_{k}\right)=\exp\{-\eta ∥{\hat{x}}_{k+1}^{*}-F\left(x_{k},u_{k}\right){∥}_{2}\},\;k\geq 0 \end{array}$$

where ${\hat{x}}_{k+1}=h^{-1}\left(z_{k+1}^{*}\right)=h^{-1}\left(A^{nat}z_{k}-Kz_{k}\right)$ and η is a hyperparameter that controls the magnitude of the reward.

### 2.3. Description of the Advantage Actor-Critic (A2C) Algorithm

This section introduces the A2C algorithm, a reinforcement learning optimization method based on the actor–critic (AC) framework [35]. The A2C algorithm differs from value-based algorithms in that it focuses on policy optimization. It combines two neural networks and employs an advantage function to assess action quality, enhancing the agent’s learning stability. The advantage function reduces variability in action selection, leading to more stable learning. Unlike the AC algorithm, the A2C algorithm incorporates an advantage function to stabilize the policy.

In the A2C algorithm, the actor selects actions based on the environment, then, using feedback from the critic, adjusts its action-selection probability. After the actor completes an action, it returns the new state and value to the critic, which computes the error and updates the action probabilities for the actor. This cycle continues until the termination condition is met.

The key formulas of the A2C algorithm are as follows. We represent the value of action *u* in a given state *x* as $Q(x_{k},u_{k})$. This paper focuses on the execution of *u* when in state s and measures the reward of the current action based on the obtained results. $V(s_{t+1})$ represents the value of the subsequent state. The relationship between functions *Q* and *V* is described by the Bellman equation, as follows:

(7) $$\begin{array}{c} Q\left(x_{k},u_{k}\right)=r\left(x_{k},u_{k}\right)+V\left(x_{k+1}\right) \end{array}$$

Denote the advantage *A* at state $x_{k}$ and $u_{k}$ as follows:

(8) $$\begin{array}{c} A\left(x_{k},u_{k}\right)=Q\left(x_{k}\right)-V\left(x_{k}\right) \end{array}$$

The advantage demonstrates the linear relationship between *Q* and *V*. This equation quantifies the advantages of an action compared with the average value in state *x*, promoting *u* balanced advantage value across the entire strategy. If the advantage value is less than 0, this action is inferior to the average and is not a good choice.

The gradient of the actor-network parameter update is calculated as follows:

(9) $$\begin{array}{c} \nabla J\left(\theta \right)=E_{\pi_{\theta }}\left[\nabla \log\pi_{\theta }\left(u|s\right)\cdot A\left(x_{t},u_{t}\right)\right] \end{array}$$

where J(θ) is the objective function of the actor network and $\pi_{\theta }$ is the probability of action output by the policy network in state *x*. $E_{\pi_{\theta }}\left[\cdot \right]$ in the formula represents the expected value when an action is selected according to the policy $\pi_{\theta }$. We update the actor-network parameters through (9).

We insert (7) into (9) and obtain the following:

$$\begin{array}{c} \nabla J\left(\theta \right)=E_{\pi_{\theta }}\left[\nabla \log\pi_{\theta }\left(u|s\right)\cdot \left(r_{t}+V\left(x_{t+1}\right)-V\left(x_{t}\right)\right)\right]. \end{array}$$

All decision values are calculated in a neural network, making the results more stable.

The steps for obtaining optimal solutions are summarized below in Algorithm 1. The optimal parameters for the neural networks are obtained using the Polyak–Ruppert averaging method, as follows: [36]:

(10) $$\omega_{N}^{★}:=\frac{1}{N-N_{0}}\sum_{n=N_{0}}^{N}\omega_{n}$$

where *N* denotes the total number of updates in the parameters and the interval $\left[0,N_{0}\right]$ with $N_{0}<N$ is known as the *burn-in* period; estimates from this period are abandoned to reduce the impact of transients in the early stages of training.
**Algorithm 1:** Closed-loop control of wound healing with A2C.    Initialize DeepMapper;
    Optimize DeepMapper with historical wound images;
    Initialize actor and critic networks;
    **while** *termination criteria not met* **do**

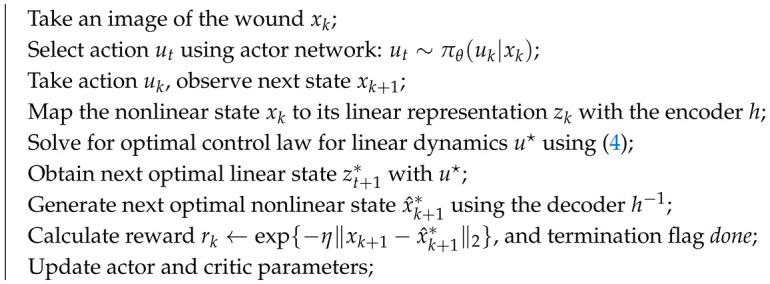

    **end**

### 2.4. Experiments in a Porcine Model

Here, we provide an overview of the in vivo experimental methods used to generate the results presented in this paper. Further details can be found in [24].

All swine experiments were conducted under the protocol approved by the University of California Davis (UC Davis) Institutional Animal Care and Use Committee (IACUC). Yorkshire mixed-breed female pigs between 70–80 lbs, approximately 4–5 months old, were used. The pigs were acclimated and trained for harness use with positive reinforcement (food treats) 7–10 days prior to the surgery (PMID: 35024682). Overnight fasting was applied with water available ad libitum before the surgery and the device change.

On the surgery day, the pigs were induced under general anesthesia, intubated endotracheally, and maintained under anesthesia using masked inhalation of isoflurane (1–5%). The back skin was clipped, depilated and prepared before being wounded. Six to eight round, full-thickness wounds (20 mm in diameter) were created on each pig. Device treatments (control or experimental devices) were administered to the cranial wounds, and the standard of care (no device, and the wounds sealed with Conformant 2 wound veil, Optifoam bolsters, and Tegaderm film dressings) was applied to the caudal wounds. The wounded area was covered with cushion foam sheets and a compression dressing/pig jacket to protect the skin of the back. A fentanyl patch as analgesic was applied for 72 h for post-operative pain management. Cycles of capturing photographs of the wounds were initiated when the device was connected to the power bank immediately after the surgery. The image data was acquired every 2 h and transmitted wirelessly to a laptop via a local Wi-Fi network for 24 h.

The in vivo experiment was conducted for 22 days, with daily wound examination and changes of the power banks to restart the device cycles. The devices were replaced on day 3 and removed on day 7 under anesthesia. Wound dressings were replaced every 3–4 days. At the endpoint of the experiment, the animals were euthanized, the devices were retrieved, and the wound tissue was harvested and bisected for histology analysis, as presented in [24].

## 3. Results

Wound-healing studies in humans have been limited by a scarcity of relevant in vivo and in vitro experimental models and the questionable practice of serial biopsies of human wounds, which are painful and may cause infection or scarring. Mouse models have an advantage in that mice are small, easy to handle, and relatively cheap. However, a high degree of phenotypic variability in skin exists between animal species, and the impact of these differences on wound healing has been recognized since at least 1915 [37]. Therefore, an accurate model of human wound healing should use an animal with skin with similar characteristics.

Porcine models have emerged as promising models in which to study wound healing, with over 1500 publications on the psychophysiology of various wound types. An advantage of using pigs is that they are anatomically and physiologically similar to humans and have been used to study many other diseases [38], such as diabetes, cardiovascular diseases, and infections. Similarities between pig and human skin also make pigs an appropriate model for the study of cutaneous wound healing. Like humans, they have a relatively thick epidermis, distinct rete pegs, dermal papillae, and dense elastic fibers in the dermis [39,40].

In this paper, we conducted one numerical study using mouse data and one experimental study in vivo with a porcine model.

### 3.1. Simulation Using Mouse Data

The mouse-image dataset used in this work was created as described in [41], and from that dataset, we generated circular-wound-only crops. The dataset includes wounds from eight mice (four from one cohort and four from another distinct cohort) that were imaged daily over sixteen days. Each mouse had two wounds, one on the left side and one on the right side. This process resulted in a total dataset of 256 images (8 mice × 2 wounds × 16 days). We split the dataset into training (224 images) and testing (32 images) subsets.

**Figure 2:**
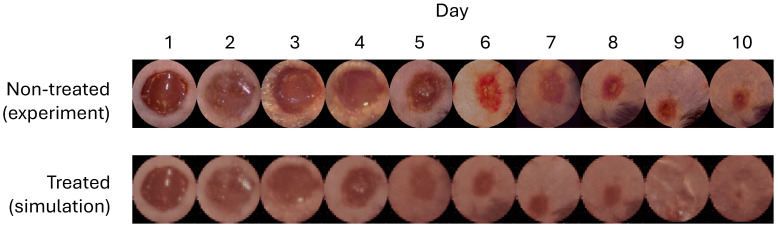
Trajectories of wound images. (**Top**): A sample trajectory of mouse-wound images in the testing dataset. It takes around 10 to 11 days for the mouse wound to heal. (**Bottom**): A sample trajectory of generated mouse-wound images using the control policy given by the DRL agents. On day 9, there were no obvious wound regions in the generated images.

An example trajectory of testing images is shown in Figure 2(top). The natural healing process of mouse wounds typically spans approximately 10 to 11 days. In our study, we employed $A^{nat}$ and $A^{act}$, as detected by the DeepMapper model. The objective was to determine whether the proposed algorithm could accelerate the wound-healing process in mice.

Since no treatment was applied to the mouse wounds during data collection, $A^{nat}$ remained unchanged. However, the components $k_{i}^{act}$ and $k_{p}^{act}$ in $A^{act}$ were modulated to new values, denoted as ${\hat{k}}_{i}^{\mathrm{act}}$ and ${\hat{k}}_{p}^{\mathrm{act}}$, respectively. This modulation was achieved via a predefined function that simulated the effects of electrical fields and fluoxetine, as described by the following equations:

$$\begin{array}{cc} {\hat{k}}_{i}^{\mathrm{act}} & =k_{i}^{act}+0.1\times \left(2*1\left\{m_{k}<=0.5\right\}-1\right)\sin\left(2\pi e_{k}\right)\\ {\hat{k}}_{p}^{\mathrm{act}} & =k_{p}^{act}+0.1\times \left(2*1\left\{m_{k}<=0.5\right\}-1\right)\sin\left(2\pi d_{k}\right) \end{array}$$

The goal was to optimize the control input $a_{k}:=\left[e_{k},d_{k}\right]$ such that the cumulative time in which the wound state $m_{k}\leq 1$ is minimized. Here, $m_{k}$ represents the state of maturation, the last stage in wound healing.

To achieve this end, we trained DRL agents to derive the control policy using the reward function defined in (6) and the values of $A^{nat}$ and $A^{act}$ predicted by DeepMapper from training images. The DRL agents were tested every 50 training episodes on the testing images, with 100 independent runs conducted using 100 different random seeds.

**Figure 3:**
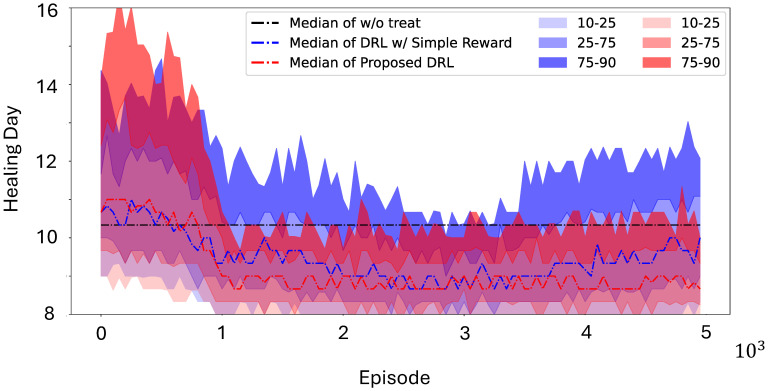
Learning curves of DRL agents over the mouse dataset with a predefined function of treatment effect, shown by percentile. Without any treatments, it would take 10.33 days for one mouse wound to heal. Our proposed method, shown in red curves, results a treatment policy that is more stable than the DRL agents implemented without the leader–follower strategy.

The results are presented in Figure 3 and show that the control policy derived by the DRL agents reduced the wound healing time to approximately 8.5 days, compared to 10.33 days for normal healing (17.71% faster). Moreover, the generated wound images in Figure 2(bottom) showed no visible wound regions by day 9, further validating the efficacy of the proposed method. Additionally, we compared this control policy with a policy optimized using a simpler reward function that directly targeted minimizing wound-healing time without employing the leader–follower tracking strategy proposed in this paper, as follows:

$$r_{k}=-1\left\{m_{k}\leq 1\right\}$$

The blue curves in Figure 3 illustrate that this alternative approach not only converged more slowly than the proposed method but also exhibited higher variance. These findings highlight the effectiveness and robustness of the proposed DRL-based control strategy in expediting the wound-healing process.

### 3.2. In Vivo Application

This work was further integrated into a 22 day in vivo porcine experiment in [24]. A total of six to ten wounds, each 20 mm in diameter, were created on the dorsum of each pig. These wounds were interfaced with a bioelectronic device with eight treatment-delivery channels: four channels were designed for fluoxetine delivery, and the rest were designed for the application of an electric field. Both the fluoxetine dosage and the intensity of the electric field were controlled via electric currents in the corresponding channels. The dosage of fluoxetine delivered from each channel was controlled by the current applied. The dosage can be calculated as follows:

$$d\left(t\right)=\int_{0}^{t}\frac{\eta g}{F\times 10^{3}}\times i\left(t\right)dt$$

where η is the pump efficiency, F is the Faraday constant, and *g* denotes the molecular weight of fluoxetine. In this application, we set η=2.2%, F= 96,485.3321 and g=309.33 g/mol. The wounds were also interfaced with a camera that took real-time images of the wound; based on these images, the proposed algorithm predicted wound stages and the DRL agent made decisions on the currents to be applied to control the dosage or intensity.

**Figure 4:**
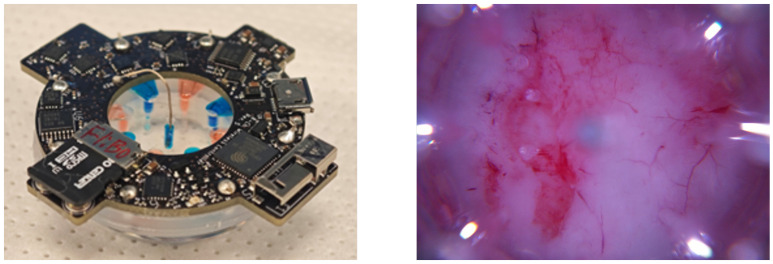
An image of EF+Flx combo actuators used in the in vivo experiments to deliver EF and fluoxetine (shown on the (**left**)) and a sample wound image captured by the device camera (shown on the (**right**)).

The real-time imaging system in [42,43] captured wound images every 2 h throughout the experiment. We fine-tuned DeepMapper to work with the device images. The wireless bioelectronic device and an image of a wound acquired by the device camera are shown in Figure 4. Note that the device images are substantially different than those taken with a standard camera, as they include wound-edge information and a different color spectrum. To preprocess the images, we subtracted [108.16, 61.49, 55.44] from the pixel values of the RGB channels in the wound image to reduce redness and downsampled the image to a specified shape (H: 128, W: 128, 3). The downsampling method we used is called the Lanczos algorithm [44]. Finally, we normalized pixel values to have zero mean and range from 0 to 1. The processed wound images were passed into DeepMapper for prediction of the wound’s healing stage and linear dynamics, based on which optimal reference signals were calculated via (4) and DeepMapper’s decoder.

**Figure 5:**
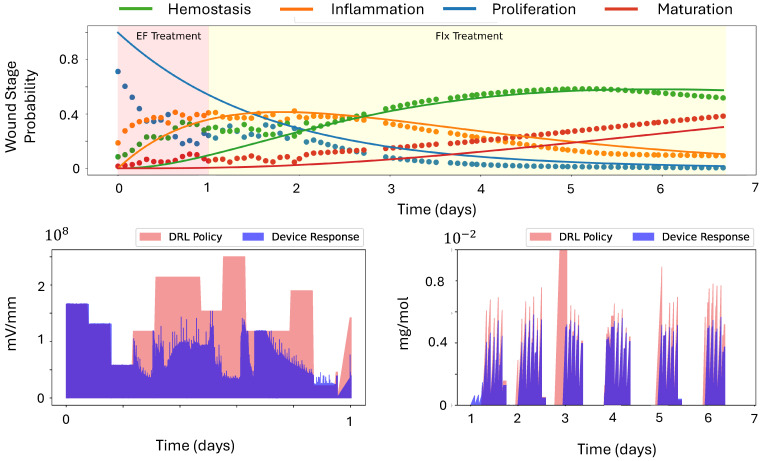
Plots of first 7-day wound-stage predictions, with the solution to the learned linear dynamics and treatment policies generated by the DRL agents across the 22-day porcine experiment. (**Top**): Wound-stage (dotted curves) predictions and solutions to predicted linear dynamics (solid curves). (**Bottom Left**): Intensity of electric field. (**Bottom Right**): Fluoxetine delivery.

Figure 5 shows the wound-stage prediction and solutions to predicted linear dynamics. In the ideal case, predicted wound stage and the solution to the linear dynamics would align with each other. However, since DeepMapper was pretrained on historical wound images, its prior knowledge about wound evolution tended to dominate early predictions, leading to deviations from the actual linear evolution. For instance, we observed a mean squared error (MSE) of approximately 69.15% in the first-day predictions. As more data were collected over time, the dotted curves in Figure 5 increasingly aligned with the linear-dynamics solutions, with the MSE reducing to 3.71% by the end of the first day.

Two DRL agents then used the wound images to make dynamic adjustments to the currents, optimizing either fluoxetine dosage or electric-field intensity so that the cumulative reward (6) was maximized. In our experiment, only one DRL agent was active at a time, meaning that when DRL agent for the electric field was active, all other channels associated with fluoxetine delivery were shut off. Initially, we used the agent controlling the electric field; once the predicted probability of inflammation reached approximately 40% and started to decrease, we switched to the DRL agent for fluoxetine delivery.

On day 0, the wounds were created. These wounds were categorized into two groups: one consisting of wounds with both an actuator and a camera and subjected to DRL treatment, and one consisting of camera-only control wounds. Devices were applied to the wounds while the pigs were under anesthesia. The devices operated in closed-loop controlled-delivery mode for 22 h each day. Power banks were replaced daily during feeding. On day 3, the devices were replaced with new ones. The DRL agent for application of the electric field was initiated on day 0, and the switch to the DRL agent for fluoxetine was made in the middle of day 1, with fluoxetine treatment continuing until day 7. The strategies used to determine the applied intensity of electric field and dosage of fluoxetine are illustrated in Figure 5.

On day 7, the devices were removed and replaced with standard-of-care treatments, and the experiment continued until harvest on day 22. At that time, the animal was sacrificed and tissue samples were collected for analysis.

**Figure 6:**
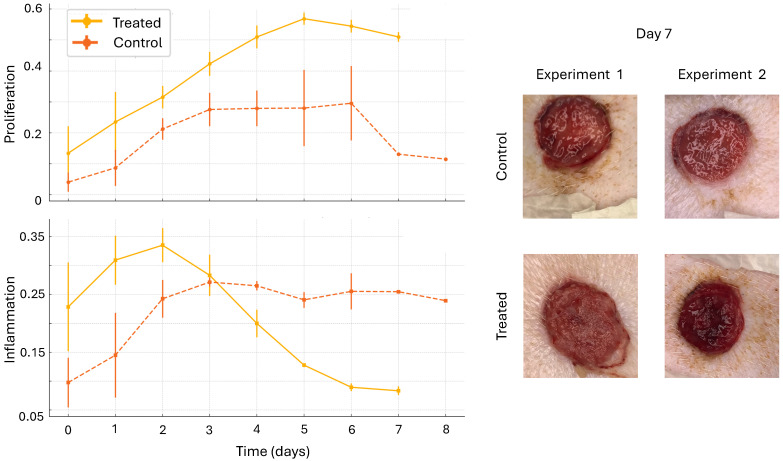
Comparison of control wounds with DRL-treated wounds. The results presented are from two experiments. In each experiment, there were two control wounds and two treated wounds. Multiple images were taken each day, and results were averaged across all wounds and experiments for each day. Missing data points are omitted. Error bars indicate one standard deviation. Images from Day 7 are shown. The bright red and shiny surface of the control wounds is an indicator of inflammation, while the softened edge of the treated wound serves as an indicator of proliferation. Thus, the DRL-based action supported the accelerated onset of inflammation and transition to proliferation.

Figure 6 shows the comparison of control wounds with DRL-treated wounds. The results presented are from two experiments. In each experiment, there were two control wounds and two treated wounds. Multiple images were taken each day, and results were averaged across all wounds and experiments for days 1–7. Images from Day 7 are shown for visual comparison and validation of the model’s predictions. The bright red, shiny surface of the control wounds is an indicator of inflammation, while the softened edge of the treated wound serves as an indicator of proliferation. Thus, the DRL-based action supported the accelerated onset of inflammation and transition to proliferation. We note that the DRL algorithm resulted in a steady and constant delivery of fluoxetine. Previous work suggests that this is an optimal strategy and that it is more effective than bolus dosing [11,12]. This result suggests that the proposed algorithm provided a treatment strategy that effectively accelerated the healing process.

## 4. Discussion

The advent of new biotechnologies has introduced an array of sensors for biological systems, addressing a critical need in medicine to integrate these sensors into closed-loop control systems. However, the inherent complexity of biological processes poses a challenge for formulating precise mathematical models. Consequently, there is a growing demand for control algorithms that can operate effectively without relying on exact models. Although sensors provide valuable insights, their measurements offer only a partial view of the underlying dynamics of real biological systems.

Wound healing exemplifies a nonlinear biological process in which different cell types play distinct roles at various stages. In our study, we assumed the availability of a sensor capable of reflecting wound stages, as suggested in [31], with the sensor data being approximately modeled by a linear system of ordinary differential equations (ODEs). Despite the convenience of using linear models, discrepancies between sensor measurements and the actual biological processes involved in wound healing reveal the limitations of linear systems for capturing the underlying nonlinear dynamics. Nevertheless, we demonstrate that nonlinear systems can be effectively monitored and controlled using observations derived from linear approximations. This approach shows promise for a wide range of nonlinear biological processes where sensors exist, but accurate mathematical models are difficult to construct.

It is also important to note that more advanced deep reinforcement learning (DRL) algorithms can further enhance control strategies and improve sampling efficiency. For instance, in large state and action spaces, Actor-Critic (A2C) methods may suffer from slow convergence and susceptibility to local minima. More advanced approaches, such as Asynchronous Advantage Actor-Critic (A3C) [34], can leverage parallelism to mitigate these issues. However, these algorithms still struggle with convergence due to the nonconvex nature of their objective functions. The integration of convex optimization techniques, as explored in [45], holds potential for overcoming this limitation.

The in vivo experimental results presented in this paper, specifically the acceleration of wound healing, were measured using predictions from DeepMapper. While our findings suggest significant differences between treated and control wounds, further studies are required. Additional experiments and data collection are necessary to fine-tune the deep learning models used in the proposed approach. Expanding the dataset, particularly to include more images of treated wounds (many of the images were from control experiments) would help strengthen the model’s accuracy and generalizability. Moreover, biological analysis of the wounds by experts is needed to substantiate the results. However, due to the prolonged nature of the wound-healing process, these experiments can be both time-consuming and expensive.

Our primary objective is to propose an adaptive learning framework that combines deep learning, optimal control, and deep reinforcement learning to accelerate wound healing. Future work will focus on designing more efficient DRL algorithms tailored to these goals.

## 5. Conclusions

In this paper, we propose an adaptive closed-loop control framework for a nonlinear dynamical system. The controller integrates deep learning, optimal control, and reinforcement learning, aiming to accelerate nonlinear biological processes such as wound healing without the need for mathematical modeling. We have demonstrated that the proposed method converges to a treatment strategy that improves wound-healing time with no mechanistic model of wound healing or knowledge about the mechanism by which the drug or EF affects biological processes.

In the future, we aim to enhance the algorithm’s performance by reducing the action space and minimizing the selection of prohibited actions, which requires integrating insight into the biology. Additionally, we will explore the application of alternative reinforcement learning algorithms to achieve significant enhancements [34,45]. Before these approaches can be considered for use in clinical practice, future work must also include safety guarantees that are wrapped around the reinforcement learning algorithm. The cost of the device is also of concern. One advantage is that there is a minimal need to monitor the device, since it has the potential to communicate wirelessly with a handheld device. For example, for the in vivo experiments, the activity of the device and acquired images can be visualized and processed in real time.

## Data Availability

All data is to be made publicly available through other referenced papers. The model and algorithm will be made available through GitHub upon publication (https://github.com/Fan-Lu/EnhancingWoundHealingUsingDRL).
